# Genome sequence of *Chryseobacterium scophthalmum LGBM4*, isolated from a red-backed salamander (*Plethodon cinereus*) in Rugar Woods, NY, USA

**DOI:** 10.1128/mra.00124-25

**Published:** 2025-04-15

**Authors:** Leslie Grossman, Brianna McLaughlin, Nana Y. D. Ankrah, Danielle E. Garneau

**Affiliations:** 1 Center for Earth and Environmental Science, State University of New York at Plattsburgh 14829, Plattsburgh, New York, USA; 2 Biological Sciences Department, State University of New York at Plattsburgh 14829, Plattsburgh, New York, USA; Indiana University, Bloomington, Indiana, USA

**Keywords:** *Chryseobacterium*, amphibian, red-backed salamander, *Plethodon cinereus*, *Chryseobacterium scophthalmum*, *Plethodon*

## Abstract

Here we present the complete genome sequence of *Chryseobacterium scophthalmum LGBM4* isolated from a red-backed salamander in Rugar Woods, Plattsburgh, NY, USA. The assembled genome comprises a 4.3 Mb chromosome with a guanine-cytosine content of 33.5%. The genome of *LGBM4* encodes several antibiotic resistance and heavy metal detoxification genes.

## ANNOUNCEMENT

Members of the *Chryseobacterium* genus are widespread in nature, occurring primarily in plant, aquatic, and soil habitats (1). *Chryseobacterium* isolates have also been associated with animals, especially amphibians infected with the pathogenic fungus *Batrachochytrium salamandrivorans* (2). In humans, *Chryseobacterium* isolates have been implicated in various clinical infections including blood and soft tissue infections (3). Most *Chryseobacterium* spp. are resistant to a wide range of antimicrobial agents, including fluoroquinolones and penicillin (4).

In this study, *Chryseobacterium scophthalmum LGBM4* was isolated from the skin of a red-backed salamander (*Plethodon cinereus*) collected from Rugar Woods, Plattsburgh, NY (44.67475 N 73.4784 W). Sterile swabs were used to collect, store, and transport salamander skin swabs. Skin swabs were inoculated on tryptic soy agar plates (Hardy Diagnostics) within 24 hours of collection and incubated at 25°C for 7 days in the dark. Isolated colonies were purified by three rounds of re-streaking to generate pure cultures.

Genomic DNA (gDNA) extraction and sequencing library preparation were completed as previously described (5, 6). Briefly, gDNA was extracted using the ZymoBIOMICS DNA/RNA Miniprep Kit (Zymo Research) from a 24-hour-old culture generated by inoculating a single bacterial colony into tryptic soy broth (Hardy Diagnostics) and incubating at 25°C without shaking. Sequencing libraries were prepared using the Rapid Barcoding Sequencing Kit v.SQK-RBK114.96 (Oxford Nanopore Technologies) according to the manufacturer’s instructions, with no fragmentation or size selection of DNA. Sequencing was conducted on the PromethION P24 platform (Oxford Nanopore Technologies), equipped with R.10.4.1 flow cells and base calling performed using Dorado v.7.1.4 on super-accurate mode (Oxford Nanopore Technologies).

Default parameters were used for all software unless otherwise specified. Reads were filtered with Filtlong v.0.2.1 (https://github.com/rrwick/Filtlong). Reads were quality filtered using a minimum Phred score of 15 and a minimum length of 1,000  bp. The genome was assembled and polished using Flye v.2.9.1 (7) and Medaka v.1.8.0 (https://github.com/nanoporetech/medaka), respectively. Assembly completeness and contamination were assessed using CheckM v.1.2.2 (8). Genome annotation was performed using NCBI Prokaryotic Genome Annotation Pipeline v.6.9 (9). A summary of assembly statistics and genome overview is provided (Table 1).

**TABLE 1:** Summary of assembly statistics and genome overview

Assembly metric	Value
Genome coverage	98×
CheckM completeness (%)	100
CheckM contamination (%)	0
Number of contigs	1
Total base pair sequenced	1,083,605,733
Total number of reads	477,533
Longest read (bp)	67,134
Read N50 (bp)	3,800
Contig N50 (bp)	4,292,624
Genome size (bp)	4,292,624
Guanine-cytosine content (%) of the genome	33.5
Genes (total)	3,927
Coding sequences (total)	3,824
Genes (coding)	3,814
rRNAs	6, 6, and 6 (5S, 16S, and 23S)
tRNAs	82
ncRNAs	3

The genome sequence of *Chryseobacterium scophthalmum LGBM4* is 4,292,624 bp (4.3 Mb), with a guanine-cytosine content of 33.5%. The genome contains 3,814 protein-coding genes and 82 tRNAs (Table 1). The genome of *Chryseobacterium scophthalmum LGBM4* encodes several genes predicted to be involved in the resistance to antibiotics and toxic compounds, including beta-lactamase, multidrug resistance efflux pumps, fluoroquinolone-resistant DNA gyrase, and copper homeostasis protein. *Chryseobacterium scophthalmum LGBM4* also encodes many genes predicted to be involved in the synthesis of antimicrobial compounds. antiSMASH v.7.1.0 (10) analysis predicted three putative secondary metabolite biosynthetic gene clusters, including regions with genes coding for nonribosomal peptide synthetase-independent, IucA/IucC-like siderophores, terpenes, arylpolyene, and resorcinol.

## Data Availability

The genome sequence of *Chryseobacterium scophthalmum LGBM4* has been deposited at DDBJ/ENA/GenBank under the accession number CP177162. The version described in this paper is CP177162.1. The associated BioProject and BioSample accession numbers are PRJNA1203911 and SAMN46004665, respectively. Raw sequence reads have been submitted to the Sequence Read Archive (accession number SRR31843741).
